# Viral Oncogenesis: Synergistic Role of Genome Integration and Persistence

**DOI:** 10.3390/v16121965

**Published:** 2024-12-23

**Authors:** Simone La Frazia, Silvia Pauciullo, Verdiana Zulian, Anna Rosa Garbuglia

**Affiliations:** 1 Department of Biology, University of Rome Tor Vergata, Via della Ricerca Scientifica 1, 00133 Rome, Italy; 2 Laboratory of Virology, National Institute for Infectious Diseases “Lazzaro Spallanzani” (IRCCS), 00149 Rome, Italy; silvia.pauciullo@inmi.it (S.P.); verdiana.zulian@inmi.it (V.Z.); annarosa.garbuglia@inmi.it (A.R.G.)

**Keywords:** persistence, episome, genome integration, latency, oncogenic viruses, papillomavirus, hepatitis B virus, Epstein–Barr virus, retrovirus

## Abstract

Persistence is a strategy used by many viruses to evade eradication by the immune system, ensuring their permanence and transmission within the host and optimizing viral fitness. During persistence, viruses can trigger various phenomena, including target organ damage, mainly due to an inflammatory state induced by infection, as well as cell proliferation and/or immortalization. In addition to immune evasion and chronic inflammation, factors contributing to viral persistence include low-level viral replication, the accumulation of viral mutants, and, most importantly, maintenance of the viral genome and reliance on viral oncoprotein production. This review focuses on the process of genome integration, which may occur at different stages of infection (e.g., HBV), during the chronic phase of infection (e.g., HPV, EBV), or as an essential part of the viral life cycle, as seen in retroviruses (HIV, HTLV-1). It also explores the close relationship between integration, persistence, and oncogenesis. Several models have been proposed to describe the genome integration process, including non-homologous recombination, looping, and microhomology models. Integration can occur either randomly or at specific genomic sites, often leading to genome destabilization. In some cases, integration results in the loss of genomic regions or impairs the regulation of oncogene and/or oncosuppressor expression, contributing to tumor development.

## 1. Introduction

Viral persistence is the ability of a virus to remain within the host for extended periods, potentially spanning the host’s entire lifetime, and persist in different cell types, which represent an important reservoir for both environmental spread and transmission to new hosts.

Persistent viral infections arise when the virus is not eradicated after an acute infection, and progression may occur in the absence of symptoms or be associated with mild (paucisymptomatic) or pronounced clinical manifestations [1].

Several viral factors play a crucial role in establishing and maintaining virus persistence in the host cell. Key factors include the absence of a cytopathic effect, the restriction of viral replication, and the maintenance of the viral genome in target cells either as episome (extrachromosomal form) or integrated into the host genome [2,3].

In addition, immune escape mechanisms, alongside genetic and epigenetic regulatory processes, enforce selective pressure that favors the outbreak of viral mutants. This process, particularly in an inflammatory environment, can be a significant part of pathogenesis and establish a favorable microenvironment for cancer development [4,5,6,7].

Latent state and/or integration of the viral genome and reliance on viral oncoprotein production represent the paramount drivers of oncogenic viruses, leading to cell immortalization and cell proliferation to optimize viral fitness [8].

The aim of this review is to provide an overview of the mechanisms driving the malignant transformation of infected cells following persistent viral infection, focusing on oncogenic viruses that integrate their genomes into that of the host cell, distinguishing between those in which integration is occasional and those in which it is obligatory, and how these differences impact oncogenic processes.

The viruses that can exist in an episomal form, typically the more frequent condition, are the Human Papillomavirus (HPV), Polyomavirus (PyV), Hepatitis B Virus (HBV), the Epstein–Barr Virus or Human Herpesvirus 4 (EBV or HHV-4), Human Cytomegalovirus (HCMV or HHV-5), Human Herpesvirus 6 (HHV-6), and Kaposi Sarcoma-associated Herpesvirus (KSHV or HHV-8) [9]. For these viruses, genome integration is generally associated with an increased risk of tumor progression [9,10,11,12,13,14,15]. The viruses that mandatorily integrate into the host genome are primarily members of the *Retroviridae* family [7,9].

Among the viruses that can exist in an episomal form, attention will be focused on three viruses of interest in human pathology belonging to different viral families: Human Papillomavirus for *Papillomaviridae*, Hepatitis B Virus for *Hepadnaviridae*, and Epstein–Barr Virus for *Herpesviridae*. Moreover, in this review, we will compare the representative members from the two main genera of *Retroviridae* that are important for human diseases: Human Immunodeficiency Virus (HIV) belongs to the *Lentivirus* genus and Human T-lymphotropic Virus type 1 (HTLV-1) belongs to the *Deltaretrovirus* genus. Despite their obligatory integration, these viruses differ significantly in their potential to induce neoplastic transformation in infected cells.

## 2. Occasional Genome Integration in Oncogenic Viruses

### 2.1. Human Papillomavirus

The Human Papillomavirus (HPV) belongs to the *Papillomaviridae* family, which includes 53 viral genera that infect a wide range of vertebrate species, from fish to mammals [16].

HPV is an icosahedral non-enveloped virus containing a double-stranded, circular, and covalently closed DNA genome of approximately 7–8 Kb. To date, 599 HPV types have been identified in humans (http://pave.niaid.nih.gov, accessed on 20 November 2024). HPVs are phylogenetically distinguished based on the sequence homology of the L1 capsid protein gene into five genera: *Alpha*, *Beta*, *Gamma*, *Mu*, and *Nu* [17].

The *Alpha-papillomaviruses* primarily infect mucosal epithelium, whereas the *Beta-* and *Gamma-papillomaviruses* mainly infect cutaneous epithelia, although they have also been isolated from oropharyngeal, anal, and cervicovaginal mucosal tissues [18]. Based on their oncogenic potential, Alpha-HPVs can be further categorized into two groups: low-risk HPV (LR) and high-risk HPV (HR) [19].

HR-HPVs are etiological agents of 5% of human cancers, causing >90% of cervical cancers in women. They are also associated with cancers of the lower genital tract, including the vulva and vagina (70%), anus (90%), penis (60%), and the oropharynx in both men and women (60%) [20,21]. According to the World Health Organization (WHO) (IARC-WHO, GLOBOCAN 2020), uterine cervical cancer represents the fourth most common cancer in women, with approximately 50% of cases caused by HPV16 [22,23]. The HPV genome comprises three functional regions. The first is the noncoding Upstream Regulatory Region (URR), also known as the Long Control Region (LCR). This region contains binding sites for the E1 (E1BS) and E2 (E2BS) proteins, essential for initiating replication, as well as binding sites for various cellular transcription factors, including specificity protein-1 (Sp1), TEF-10, activator protein-1 (AP1), and NF1. The second region is the “early region”, which includes several open reading frames (ORFs) such as E1, E2, E4, E5, E6, and E7. The E1 and E2 proteins play a crucial role in the integration process, while E5 is involved in regulating cell proliferation and apoptosis, though it is not present in all HPV types. E6 and E7, also known as “oncoproteins”, are critical for neoplastic transformation [24] and are overexpressed in HPV-related tumors. The “late regions” (L), L1 and L2, encode two structural proteins that form the viral capsid [25]. HPV infection is highly common in humans, with an estimated 80% of women experiencing an HPV infection in the first half of their lives; however, over 90% of these infections resolve spontaneously within 5–7 years due to the host immune response [21]. Infections are often subclinical or asymptomatic, although they may manifest as benign warts or papillomas on the skin or genital areas. Due to its frequent presence on both mucosal surfaces and skin, HPV is considered a commensal component of the microbiota, particularly on the skin [26].

The immune response, both innate and cell-mediated, can lead to the eradication of HPV infection, or alternatively, a persistent latent infection may develop, which, over the years, can result in the neoplastic transformation of infected cells. In the basal and parabasal cell layers, the E1 and E2 proteins initiate the viral replication cycle. E2 serves as the primary transcriptional regulator of the virus, while E1 acts as a helicase. These proteins bind the replication origin, promoting viral DNA synthesis [27]. The HPV genome is amplified and maintained in an episomal form, with approximately 100–200 copies per cell [episome copies]. Additionally, E1 can induce DNA damage, and with the activation of the DNA damage response (DDR), a reduction in cellular proliferation may be observed [28]. E2 also has the ability to downregulate early genes, including those encoding the E6 and E7 proteins, by binding to the viral noncoding LCR and blocking the binding of transcription factors necessary for the expression of these proteins [29].

The E5 protein promotes immune evasion and reduces the dependence of growth factors on cells, resulting in enhanced cell proliferation [30]. E5 interferes with the host immune response by impairing major histocompatibility complex (MHC) class I and II levels, preventing the efficient acidification of late endosomes via modulation of H^+^-ATPase [31]. E5 also plays a role as an inhibitor of interferon-I (IFN-I) signaling pathways, reducing the amount of peptides exposed to the immune system. Furthermore, E5 has been found to bind directly to the mitochondrial antiviral signaling protein (MAVS) and stimulator of interferon genes (STING) proteins, which are key proteins that block their activity and hamper the immune response against tumor cells [32,33].

E5 upregulates EGFR (Epidermal Growth Factor Receptor) and prevents its degradation, promoting cell growth and survival, especially in HR strains such as HPV16 [34,35].

Moreover, the HPV16 E5 oncoprotein inhibits interferon signaling in keratinocytes, specifically IFN-κ, thereby supporting episomal viral maintenance [36].

The E6 and E7 proteins interact with p53 and pRb-E2F, respectively, both of which play essential roles in cell cycle checkpoint regulation [37,38]. Additionally, HR E6 recruits the ubiquitin-protein ligase E6AP, inducing the proteasome-dependent degradation of the tumor suppressor p53 and the mitotic kinesin CENP-E, which promotes cell proliferation and chromosomal instability, respectively [39,40,41,42]. Moreover, p53 expression can also be suppressed at the transcriptional level via inhibition of the recruitment of CREB-binding protein (CBP) and p300 transcription factors to the p53 gene promoter by HR E6 [43]. The E6 protein activates human telomerase reverse transcriptase (hTERT), stabilizing telomeres and preventing cellular senescence [44]. Another key pro-oncogenic role of HR-HPV E6 is its ability to interact with and degrade PDZ-domain-containing proteins, including Membrane-Associated Guanylate Kinase-1 (MAGI1), Discs large homolog 1 and 4 (DLG1, DLG4), Scribble (SCRIB), and Protein Tyrosine Phosphatase Non-Receptor Type 13 (PTPN13). These proteins are involved in signal transduction, cell polarity, and adhesion; their loss is often associated with tumorigenesis [45].

Moreover, HR E6 upregulates the Apolipoprotein B mRNA editing enzyme, catalytic polypeptide (APOBEC), which is part of the editosome involved in mRNA modifications through cytidine-to-uracil deamination, promoting the onset of viral mutants with enhanced potential for immune escape and cell tumor progression [46].

The HR E7 protein plays a pivotal role in the immortalization and tumor transformation of infected cells by binding to the retinoblastoma protein (pRb), a tumor suppressor, causing its dissociation from the E2F transcription factor. This results in bypassing the G1 checkpoint, premature entry into the S phase of the cell cycle, and genomic instability [47]. However, the interaction between HR E7 and pRb alone does not fully explain this viral oncoprotein ability to immortalize and transform cells.

HR E7 also promotes the ubiquitin–proteasome-dependent degradation of the non-receptor protein tyrosine phosphatase PTPN14 through the E3 ubiquitin-protein ligase component N-Recognin 4 (UBR4), with reduced PTPN14 levels linked to an increased risk of carcinogenesis [48]. Moreover, there is a cooperation between the E6 and E7 proteins, which regulate the expression of HER3. The overexpression of HER3 is associated with poor prognosis in cervical carcinoma [49]. Furthermore, the HPV16 E6 and E7 proteins induce the epithelial-to-mesenchymal transition (EMT) signaling pathway via FGF-2 and FGF-4, contributing to the development of invasive cancer [50]. Additionally, the pro-angiogenic factors HIF-1 alpha and VEGF are upregulated by the HPV-16 E6 and E7 oncoproteins, thereby promoting the angiogenic process in cervical carcinoma cells [51,52].

HPV-16 infections tend to persist longer than infections with other HPV types. However, persistence alone is not sufficient for progression to high-grade dysplasia. Differences in the progression potential between HPV types are linked to the biological activities of E6 and E7, which may explain the higher likelihood of developing more severe lesions with high-risk HPV types.

A consequence of persistent HR-HPV infections is the integration of the viral genome into the host genome [53]. However, the integration of the HPV genome into the host genome is not observed in all HPV-associated tumors. For instance, HPV genome integration has been observed in 80% of HPV-positive cervical tumors [54], while the integration frequency is lower in HPV-positive laryngeal carcinoma than in cervical carcinoma [55]. Different HPV genotypes also exhibit varying integration frequencies: HPV18 is integrated in 100% of cervical cancer cases, whereas HPV16 is integrated in only 80% of cases [54]. This suggests that a small percentage of HPV-related cancers can develop without viral genome integration into the host genome, as high expression levels of E6 and E7 oncoproteins alone may be sufficient for cellular transformation [56]. In general, in HR-HPV infections, the episomal form of the viral genome is frequently observed in cervical intraepithelial neoplasia (CIN) CIN1 and CIN2, whereas HPV genome integration into the host genome is more commonly found in CIN3 and invasive carcinoma [57]. The E2 ORF region is often the preferred site of integration, which results in the loss of E2 functionality. Consequently, E2 can no longer downregulate E6 and E7 expression, leading to an increase in their transforming potential [58]. Additionally, the potential loss of E5 is implicated in genome stability and is another driver of genome integration [36].

However, integration into the host genome also leads to alterations in cellular gene expression and genomic and chromosomal instability at HPV DNA integration sites. High levels of E6 and E7 expression may further induce polyploidy due to the deregulation of cellular genes that typically control the G2M phase transition to mitosis [59]. E6 and E7 are also considered responsible for telomere lengthening, resulting in altered chromosome segregation [60]. Methylation appears to be a significant factor in the HPV integration process, as it can lead to changes in gene expression and contribute to genomic instability, favoring integration [61].

The integration process can be divided into several steps. The first step involves a break in the circular DNA, which linearizes the HPV genome. HPV DNA can integrate as a single copy or multiple viral DNA segments may be dispersed across various sites in the host cell genome (integration events). Integration typically occurs in transcriptionally active chromatin regions [62]. Several theories have been proposed to explain the integration phenomenon. One suggests that integration occurs accidentally during the repair of double-stranded DNA breaks [63].

Notably, the E6 and E7 genes are rarely disrupted during integration, while breakpoints frequently occur within the E1 and E2 coding regions. Episomal replication does not appear to be involved in the integration process [64]. Deletions, duplications, and translocations are often present in regions flanking HPV integration sites [65].

**Figure 1:**
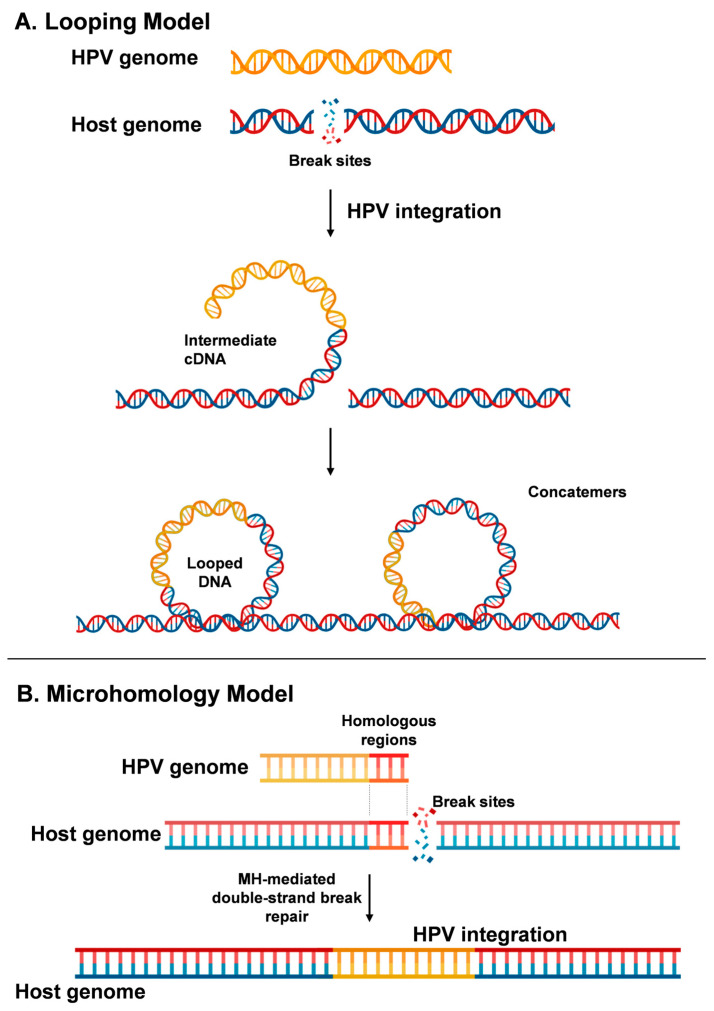
Proposed models for HPV integration process into the host genome. (**A**) The *Looping Model* suggests that the viral DNA forms loop structures, facilitating its integration into the host genome through breakage and subsequent repair mechanisms at the integration site. (**B**) The *Microhomology Model* proposes that integration occurs via regions of microhomology (MH) between the viral and host DNA, enabling alignment and recombination during repair processes.

**Figure 2:**
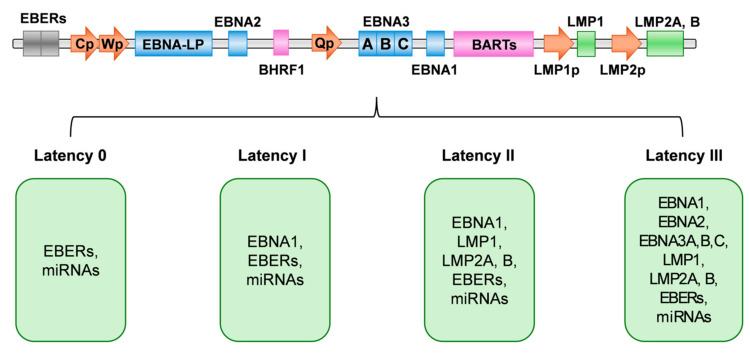
Schematic representation of the Epstein–Barr Virus (EBV) genome and associated latency programs. The EBV genome is illustrated, highlighting key regions and genes involved in viral latency. Latency states are characterized by distinct expression profiles, critical for the virus’s ability to evade immune responses and establish persistent infection. Abbreviations: EBERs, EBV-encoded small RNAs; Cp, C promoter; Wp, W promoter; EBNA-LP, EBV Nuclear Antigen-Leader Protein; EBNA2, EBV Nuclear Antigen 2; BHRF1, BamHI fragment H rightward open reading frame 1; Qp, Q promoter; EBNA3, EBV Nuclear Antigen 3; EBNA1, EBV Nuclear Antigen 1; BARTs, BamHI-A Rightwards Transcripts; LMP1, Latent Membrane Protein 1; LMP1p, LMP1 promoter; LMP2A, B, Latent Membrane Protein 2A, B; LMP2p, LMP2 promoter.

Two models have been proposed to describe the integration process: (1) the *Looping Model*, which suggests that HPV integrates by forming a non-contiguous bridge between human DNA at the two double-stranded break sites [65] (Figure 1A), and (2) the *Microhomology Model*, which proposes interactions between homologous regions of HPV and human DNA break sites (Figure 1B).

Another hotspot for HR-HPV integration is the *MACROD2* gene (Microsomal Antioxidant-Related 2), which is implicated in maintaining genomic stability, particularly concerning DNA repair and cellular responses to oxidative stress. The integration of the HR-HPV DNA into the *MACROD2* gene may be a hallmark of cancer cells. Overall, the different integration hotspots could represent predictive markers of disease progression aimed at assessing the risk of malignancy in individuals with HPV infection [66].

Moreover, studies in transgenic mice with an integrated HPV16 genome have demonstrated distinct HPV pattern expression in different cell types. For instance, Yang et al. observed that the E2 gene was expressed only in T-cell lymphomas, while E6 and E7 proteins were expressed in both carcinoma cells and T-cell lymphomas [67]. Recently, the absence of viral genome integration and aberrant episomal replication leading to rearranged, mutated, and dimeric episomes overexpressing E6 and E7 have been observed in at least 30% of HPV-16 tumors [68].

### 2.2. Hepatitis B Virus

To date, Hepatitis B Virus (HBV) is still a major public health problem, with approximately 254 million people chronically infected, 1.2 million new cases, and an estimated 1.1 million deaths in 2022 (www.who.int/news-room/fact-sheets/detail/hepatitis-b, accessed on 17 September 2024).

Transmission occurs through parenteral and sexual routes, and after an incubation period of 30–180 days, acute infection can develop. This infection may range from asymptomatic to acute viral hepatitis, varying from mild to severe, and, in rare cases, it may be fatal. When HBV infection occurs in adulthood, less than 5% of cases progress to chronic hepatitis. In contrast, if infection takes place during childhood, approximately 95% of cases become chronic [69]. Chronic HBV infection can lead to liver cirrhosis and hepatocellular carcinoma (HCC) after many years [70].

Regarding the virion structure, HBV is an icosahedral enveloped virus containing a small, partially double-stranded circular DNA genome of 3200 base pairs (bp) belonging to the family *Hepadnaviridae*. The viral polymerase is covalently attached to the 5′ end of the (−) strand and possesses DNA- and RNA-dependent DNA polymerase activity, acting as a reverse transcriptase in the latter function [71]. The *Hepadnaviridae* family is characterized by viruses with limited host range and tissue tropism [72].

The HBV genome consists of four overlapping open reading frames (ORFs):

(1) ORF S encodes the envelope proteins, expressed as three proteins of varying lengths from a single open reading frame, large (pre-S1, pre-S2, and S), middle (pre-S2 e S), and small surface (S) proteins [73], collectively named Australian antigen (HBsAg). Mutations in the pre-S region increase in chronic stages and are prevalent in patients with cirrhosis and HCC [74].
(2) ORF P encodes the polymerase (P), which functions as a reverse transcriptase (RT).
(3) ORF C encodes the core (C) protein and the pre-core (pre-C) protein, known as the Core antigen (HBcAg) and E antigen (HBeAg), respectively.
(4) ORF X encodes the X protein, which functions as a transactivator and is involved in tumorigenic processes [75]. The HBx protein plays a crucial role in the normal HBV cycle. It helps overcome host restriction factors that might inhibit viral replication [76,77]. Additionally, HBx facilitates HBV transcription by promoting the acetylation of histones, which is essential for efficient transcription from cccDNA [78,79].

The double-stranded DNA of HBV exists in two forms: relaxed circular DNA (rcDNA), which is generally present in approximately 90% of virions [80], and double-stranded linear DNA (dslDNA), found in about 10% of virions. The linear double-stranded DNA is replication-defective but can integrate into hepatocyte chromosomes through non-homologous recombination.

The replication phase of HBV involves several steps. Upon entry into the cell, rcDNA is transported to the nucleus, where it is converted into covalently closed circular DNA (cccDNA). This cccDNA serves as a template for producing viral transcripts, including pre-genomic RNA (pgRNA), which is subsequently reverse transcribed into rcDNA and encapsidated into a new virion. However, a small amount of dslDNA is also generated, which can be encapsidated into virions and, upon infecting a new cell, may integrate into the host genome at double-stranded DNA breaks [81].

The mechanisms driving HBV persistence and the development of HCC following viral genome integration involve chromosomal instability, insertional mutagenesis (which upregulates oncogenes or silences tumor suppressors), and the expression of mutated viral proteins [82].

The integrated DNA of HBV is defective because it cannot replicate, as viral transcripts for the C protein and RT are not produced, and, most importantly, the pgRNA is not generated [83]; however, it can still express viral proteins, such as the surface proteins (HBsAg) and the X protein. These proteins, L, M, S, and X, are truncated or chimeric due to the use of a viral polyadenylation signal located upstream or a cellular polyadenylation signal downstream of the conventional one [84].

In 23% of HCC cases, a chimeric RNA transcript is formed from human Long Interspersed Nuclear Elements 1 (LINE1) and the viral HBV-X genes. This chimeric RNA can sequester miR-122, promoting abnormal mitosis in liver cells [85].

Patients with HBX forms lacking the C-terminal domain are associated with a worse prognosis.

From a molecular standpoint, this is due to the suppression of the gene for thioredoxin-interacting protein (TXNIP), which acts as a negative regulator of glucose metabolism during HCC progression [86]. In vitro models have shown that integration does not occur continuously, so the multiple integrations observed in hepatocellular carcinomas are likely due to new infections [87].

The non-homologous end-joining (NHEJ) DNA repair pathway, which is error-prone, plays a role in HBV integration [88]. This phenomenon associated with HBV integration is also known as “insertional mutagenesis”, characterized by insertions and deletions at the integration sites, leading to alterations in genes involved in cell proliferation, the upregulation of oncogenes, or the inactivation of tumor suppressor genes [82].

HBV integration has been shown to be nonclonal in non-tumorigenic processes [89], resulting from random events, whereas in HCC, HBV fragments are clonally integrated near genes involved in carcinogenesis [90]. Viral genome integration has been primarily observed in patients with high viremia, but recent studies have demonstrated its presence even in patients with moderate or low viral DNA levels [15,91].

Several studies have shown that in HCC, the hotspot for HBV integration is near the h-TERT gene, which is associated with a poor prognosis in most cases. Other notable integration sites for viral DNA include the histone gene of methyltransferase (KMT2B), the glioma-associated oncogene family zinc finger 2 (GLI2), and the cyclins A2 (CCNA2), D1 (CCND1), and E (CCNE1), as well as Mixed Lineage Leukemia 4 (MML4) proteins, which are important for cell cycle regulation [82,89,90,92,93,94,95].

HBV integration can lead to gene rearrangement, variations in the number of chromosomes in the host genome, or interchromosomal fusions. Among the most common features are the loss of tumor suppressor p53 activity, amplification of oncogenes such as TERT and MYC [96], and telomeric deletion [97]. Approximately 90% of HBV-related HCC cells contain integrated HBV DNA at multiple chromosomal sites [96,98,99].

The phenomenon of HBV integration can occur at any stage of infection [100], meaning that clinical improvement does not necessarily indicate complete resolution of HBV infection, considering that both cccDNA and the integrated genome can persist in the nucleus of hepatocytes [101,102].

The development of HCC following chronic persistent HBV infection can result from direct mechanisms related to the viral replication cycle or, alternatively, more often in association with indirect mechanisms driven by inflammation and the immune response against infected hepatocytes or HBV DNA integration [81,102,103].

Another direct mechanism promoting liver cancer by HBV is the expression of specific viral proteins, the most important of which is the X protein. HBX plays multiple roles, promoting chronic inflammation, oxidative stress, and genomic instability, thereby accelerating cancer progression and contributing to tumor cell invasion and survival.

HBX is involved in epigenetic changes such as DNA methylation, histone acetylation, and miRNA expression [104].

The HBX protein can interfere with fundamental cellular processes by modulating various signaling pathways, including Mitogen-Activated Protein Kinase/Extracellular Signal-Regulated Kinase (MAPK/ERK), Phosphoinositide 3-Kinase/Protein Kinase B- Mechanistic Target of Rapamycin (PI3K/AKT-mTOR), Janus Kinase/Signal Transducer and Activator of Transcription (JAK/STAT), Wnt/β-catenin, Nuclear Factor Kappa-Light-Chain-Enhancer of Activated B-Cells (NF-kB), Transforming Growth Factor Beta (TGF-β), and cyclin-dependent kinase (CDK), p53, and pRb, all of which are involved in the regulation of gene expression, autophagy, apoptosis, cell proliferation, and survival [82,105,106]. Interestingly, in transgenic mice with HBV knock-out for the p65 subunit of NF-κB, the inflammatory responses are impaired, survival signals in hepatocytes are diminished, and the incidence of HCC is reduced, highlighting the importance of this signaling pathway in HBX-mediated malignant transformation of hepatocytes [105,107]. Moreover, in truncated forms of HBX, in addition to the suppression of Thioredoxin-interacting protein (TXNIP) gene expression, apoptosis inhibition is also associated, which are events that increase the risk of hepatocarcinogenesis [15].

In the murine model, overexpression of the X protein was associated with the development of HCC, although the X protein alone was not sufficient to initiate the neoplastic transformation process, and additional DNA damage in the host cell was required for transformation to occur [108,109]. Moreover, high expression of HBsAg can promote neoplastic transformation, but this is typically observed in association with increased albumin production [110].

During viral infection, oncoprotein HBX induces Smc5/6 (Structural maintenance of chromosomes protein 5/6) complex degradation, impairing DNA damage repair and promoting tumor progression [111]. Moreover, HBX upregulates DNA methyltransferase 1 (DNMT1) and Helicase DDX5, which is involved in improved risk of HCC and metastasis [112,113].

In the case of HBsAg, truncated forms retained inside the hepatocyte can potentially dysregulate apoptosis and exacerbate the abnormal proliferation in infected cells [114]. For example, preS1 and preS2 deletions contribute to cancer transformation in glass hepatocytes or the expression of truncated forms of HBsAg. These mutants regulate the transforming growth factor-β/small mother against the decapentaplegic (TGF-β/SMAD) signaling pathway, thereby increasing the risk of HCC [115,116].

In a murine model mimicking chronic HBV infection, transgenic mice expressing the viral L protein at low levels were asymptomatic. However, when their immune system was replaced with cells from non-transgenic syngeneic mice, they developed liver damage and HCC. This suggests the importance of the immune response in eliminating infected cells that could progress to malignant transformation, as well as in the emergence of viral mutants with higher oncogenic potential [117].

Regarding the indirect mechanisms that may contribute to HCC development in chronic HBV infections, we can include immune evasion (both innate and adaptive), production of reactive oxygen species (ROS), and pro-inflammatory cytokines, all of which lead to chronic inflammation.

In particular, CD8+ T-cells are highly activated during chronic infection and induce an exacerbated inflammatory response that creates the conditions for the development of liver cirrhosis and HCC [118].

The cellular protein apolipoprotein B mRNA-editing enzyme catalytic polypeptide-like protein 3 (APOBEC3) is a deaminase that induces a high mutation rate in the HBV genome, rendering virions replication-defective and thus hindering infection progression and the establishment of viral persistence. In a case–control study involving a Moroccan population, individuals with allelic deletion variants of APOBEC3 exhibited significantly higher viral loads and faster disease progression compared to control subjects, whereas no differences were observed concerning susceptibility to chronicity [119]. This observation was further supported by an in vivo study of HBV infection in tree shrews (*Tupaia belangeri*), which can exhibit clinical outcomes similar to those in humans. The study demonstrated that APOBEC3 in tree shrew limits HBV replication and persistence more effectively than in human infections [120].

### 2.3. Epstein–Barr Virus

Epstein–Barr virus (EBV), or Human Herpesvirus 4 (HHV-4), belonging to the family *Herpesviridae*, subfamily *Gammaherpesvirinae*, has a marked tropism for B lymphocytes and epithelial cells. EBV is an enveloped double-stranded DNA virus enclosed in an icosahedral capsid. The genome of ~172 Kbp encodes for ~80 proteins and 46 functional small untranslated RNAs involved in lytic cycle and latency [121].

The EBV gp350/220 protein mediates adsorption to Complement Receptors (CRs) CD21 present on B lymphocytes, followed by the binding of EBV gp42 to Human Leukocyte Antigen II (HLA-II), which results in a conformational change in EBV gB and the EBV gH/gL complex with the consequent fusion of the viral envelope with the host cell plasma membrane [122]. In B-cells, EBV establishes latency, but under specific stimuli, it can activate the lytic cycle (reactivation) with temporally regulated gene expression involving the sequential transcription of immediate early (IE), early (E), and late (L) EBV genes, typical of *Herpesviridae* [123]. The cellular transcriptional factors bind the IE BamHI Z fragment L region gene 1 (BZLF1) and BamHI-R fragment L region gene 1 (BRLF1) promoters driving the reactivation state [124]. They encode for the transactivator proteins Z (ZEBRA) and R (RTA) responsible for the expression of early genes involved in viral DNA replication [125]. Replication of the viral genome occurs as extrachromosomal DNA (episome) independently of cellular DNA replication. Finally, after replication of the viral genome, late proteins (L) involved in viral morphogenesis are produced.

In latent infection, a restricted set of genes different from those expressed during the lytic cycle is transcribed. In relation to latency genes, the EBV genome encodes for nine proteins (Epstein–Barr Nuclear Antigens, EBNAs, and Latency Membrane Proteins, LMPs), two Epstein–Barr virus (EBV)-encoded small RNAs (EBERs), and 44 miRNAs generated from the BamHI-A Rightwards Transcripts (BARTs). The genes of the six EBNAs (EBNA-LP, EBNA1, EBNA2, EBNA3A, EBNA3B, and EBNA3C) and the three LMPs (LMP1, LMP2A, and LMP2B) are transcribed from five different promoters (Cp, Wp, Qp, LMP1p, and LMP2p) [126] (Figure 2).

Four latency programs were observed in EBV-infected B-cells, from 0 to III type, depending on the viral protein expression profiles, during which BZLF1 and BRLD1 were not expressed and the lytic cycle was suppressed [127]. Noncoding RNAs (ncRNAs) are expressed in all latency programs [128]. Underlying the establishment and maintenance of a specific latency program are epigenetic mechanisms driving the methylation of specific regions of viral DNA [129]. Soon after EBV entry into B-cells, the latency III program is established, characterized by the expression of all latent proteins with B-cell transformation or immortalization. The EBV genome is maintained in an episomal form in proliferating B lymphocytes by regulation at a specific latency-associated origin of replication (ori P) [130]. From the two early promoters Wp and Cp, whose activation is finely regulated, mRNAs coding for the six EBNAs proteins are transcribed [131]. EBNA-LP, EBNA1, and EBNA2 bind the Cp promoter, leading to upregulation of EBNA mRNAs, whereas EBNA3A,B,C act as negative regulators [130]. EBNA2 and EBNA-LP transactive the LMP promoters to produce the LMP1, LMP2A, and LMP2B proteins [132,133].

Upregulation of DNMT3B (DNA methyltransferase 3 Beta) in germinal center B-cells causes methylation of the Cp promoter, leading to the switch from latency III to latency II status [134]. Latency II is characterized by the expression of LMPs and of EBNA1 from the Qp promoter, whereas the block of Cp activation does not allow the expression of the other EBNA proteins [135,136].

In proliferating B-memory cells, downregulation of LMP1 and LMP2 expression promotes the transition from latency II to latency I, where only EBNA1 is expressed, whereas no latency proteins (latency 0) are expressed when the cells become resting [136,137].

Transmission occurs via the airway, and latent infections occur in 90% of the adult population [138].

EBV is most widely known for its association with infectious mononucleosis, a mild, self-limiting lymphoproliferative disease due to polyclonal expansion of B lymphocytes. However, EBV is particularly involved in the malignant transformation of epithelial and lymphoid cells, playing a key role in the development of various tumors, including B- and T-lymphomas (Burkitt’s lymphoma, Hodgkin’s lymphoma, diffuse large B-cell lymphoma, systemic EBV-positive T-cell lymphoma, and natural killer/T-cell lymphoma) and nasopharyngeal (NPC) and gastric carcinomas (GC) [13,20,139].

In particular, EBV is involved in 95% of Burkitt’s lymphomas (BLs) in association with people having *Plasmodium falciparum* co-infection in geographic areas where malaria is endemic [140]. In addition to the endemic form of BL, the WHO has classified two other forms, sporadic and human immunodeficiency virus (HIV), associated with BLs, where EBV is found in 30–40% and 5–10% cases, respectively [13].

EBV occurs in different latency states depending on the type of tumor. In BL Burkitt’s lymphoma, we have latency state I, and in Hodgkin’s lymphoma and nasopharyngeal carcinoma, latency state II [129]. A particular latency state, named type IIb, has been observed in EBV infectious mononucleosis, where EBNAs are highly expressed, while LMPs and lytic cycle genes are expressed at low levels [137].

The role of latent protein and nc-RNAs is paramount in the carcinogenesis process.

EBNA-1 is the only protein expressed in all latency states and, therefore, is essential for viral persistence, which is maintained by modulating expression levels so that it is sufficient to sustain the latency state but not such that EBNA1-positive cells become targets of the immune response [141]. Moreover, EBNA1 promotes the phosphorylation and degradation of promyelocytic leukemia (PML) proteins in the nuclear bodies (NBs) by casein kinase 2 (CK2) and ubiquitin-specific protease 7 (USP7), respectively, leading to lytic infection [142].

EBNA2 interacts with Early B-cell factor 1 (EBF1), promoting MYC expression that leads to S-phase cell cycle progression [143].

EBNA3A and EBNA3C are involved in B-cell differentiation and transformation. EBNA3C inhibits p53 by binding to it and interacting with inhibitor of growth 4 (ING4) and ING5. It leads to the degradation of cell cycle regulators pRb and p27 and modulates the activity of cyclins A, D1, and E [144,145].

LMP1 triggers several signaling pathways, such as NFκB, mitogen-activated protein kinase (MAPK), phosphatidylinositol 3-kinase (PI3K)/Akt, and c-Jun N-terminal kinase (JNK), driving apoptosis, proliferation, and transformation to promote NPC [146].

LMP2A possesses a role in anti-apoptotic and transformation in Hodgkin lymphoma tumors [147]. LMP2B is involved in the induction of the lytic cycle [148].

EBER1 and EBER2, transcribed by host RNA polymerase III, play a crucial role in the host innate immunity by blocking protein kinase RNA-dependent (PKR) activation, inducing type-I interferon (IFN)s and interleukin 10 (IL-10), and promoting oncogenesis [149,150,151].

BART lncRNAs impair gene expression involved in cell adhesion, inflammation, and immunity by the epigenetic regulation of gene expression [152,153,154].

EBV represents a viral model in which the DNA genome is present in the nucleus of the host cell, in most cases as extrachromosomal episomes and in smaller percentages (0.1–1% of infected hepatocytes) integrated into the human genome [13,155]. Comparing EBV with other oncogenic viruses such as HPV and HBV, it was observed that EBV has no integration of hotspots in the cellular genome; however, in BL cells, there are multiple sites, but in a non-random pattern, with integration in fragile regions occurring during DNA damage repair that, when occurring upstream of proto-oncogenes such as MYC or within tumor suppressor genes, increase the risk of developing tumors [156].

EBV integration is common in latency cell reservoirs and contributes to long-term viral persistence.

The integration of the EBV genome into host DNA involves the formation of double-strand breaks (DSBs) in viral DNA, which are primarily repaired by the host DNA repair machinery through the classical non-homologous end-joining (NHEJ) pathway, which enables DNA ends to be joined even in the absence of significant sequence homology, facilitating the integration of exogenous DNA such as the viral genome [157]. However, microhomology sequences at integration sites, such as insertions of 2–10 base pairs, are often used by the NHEJ mechanism to optimize alignment and binding, increasing the efficiency of integration [157].

EBV integration primarily occurs in the transcriptionally inactive heterochromatic regions of the host genome, a strategy that helps the virus evade immune detection. Breakpoints of EBV integration are distributed throughout the viral genome, particularly near the origin of replication (oriP) and terminal repeats, but not within the long internal repeats, suggesting that integration is associated with replication of the viral genome. The integration sites show strong colocalization with CpG islands and CTCF binding sites, suggesting that EBV may exploit the functions of CCCTC-binding factor (CTCF), a zinc-finger protein involved in chromatin architecture and transcriptional regulation, to facilitate integration [156]. To promote integration, EBV takes advantage of several DNA repair factors, including the catalytic subunit of DNA-dependent protein kinase (DNA-PKcs) and Ku70/80, which bind to DNA ends at DSBs [13,157,158].

In cancers such as BL, NPC, and Hodgkin’s lymphoma, EBV integration contributes to genomic instability, including chromosomal rearrangements, gene amplification, and translocations, playing a pivotal role in disease progression in both primary and metastatic tumors [159,160].

In addition, EBV can coexist as a mixture of episomal and integrated EBV-DNA in some cells, accelerating the malignant transformation of infected cells and tumor development [13].

## 3. Comparative Analysis of HTLV-1 and HIV: Tumorigenic Potential Linked to Replication and Cell Proliferation

### 3.1. Human T Lymphotropic Virus Type 1

The Human T Lymphotropic Virus Type 1 (HTLV-1), belonging to *Deltaretrovirus* genus, has a similar genome and virion structure to HIV, as described below. A significant difference in the viral particle of HTLV-1, in comparison with HIV, is that the genome is enclosed in an icosahedral capsid [161,162]. The 8.5–9 kb genome encodes for three polypeptide precursors, Gag, Pol, and Env; for two regulatory proteins, Tax (p40) and Rex (p27); for p21Rex and HBZ (HTLV-1 Basic Leucine Zipper Factor), and four other accessory proteins, p8, p12, p13 and p30 [163]. Gag cleavage produces capsid (CA, p24), nucleocapsid (NC, p15), and matrix (MA, p19) proteins; Pol cleavage gives rise to reverse transcriptase plus RNAse-H (RT, p62/p51), integrase (INT, p98), and protease (PR); and Env cleavage produces the surface protein (gp46) and transmembrane protein (gp21), used as VAP and fusion protein, respectively. Tax and Rex of HTLV-1 are analogous to Tat and Rev of HIV-1, respectively [162]. The proviral DNA of HTLV-1 obtained by reverse transcription, as in HIV, has two long terminal repeat (LTR) sequences at its ends [161,162]. HTLV-1 contains two unique regions, U3 and U5, and one repeat (R) within each LTR. Subsequently, viral integrase promotes the integration of proviral DNA into the host genome, a critical step in the virus replication cycle. The mechanism of viral genome integration is described in detail in the HIV section, as it is common to all retroviruses and best characterized. HTLV-1 integration is non-random and occurs at specific sites in the host genome, predominantly in transcriptionally active regions. This preference for integration near genes involved in cell growth and survival may contribute to long-term persistence, activation of host genes, and cellular transformation. HTLV-1-infected cells proliferate in vivo, forming clonal populations defined by unique proviral integration sites [164]. The virus, predominantly found in CD4+ T lymphocytes, can undergo mitotic or infectious replication. Mitotic replication occurs when proviral DNA is duplicated during the division of infected cells, while infectious replication involves the transcription of proviral DNA into mRNAs for viral proteins and as the template for new viral genomes, ultimately leading to the production of new infectious particles [165]. Unlike HIV, HTLV-1 requires lymphocytes in active cell division to replicate efficiently, correlating with its association with the occurrence of lymphomas.

HTLV-1 transmission can occur via a unique viral biofilm mechanism, characterized by its ability to form extracellular, carbohydrate-rich biofilm-like structures that enhance infection efficiency by concentrating viral particles near host cells [166,167]. Additionally, HTLV-1 transmits virions between CD4+ cells through virological synapses (single or polysynaptic) with higher efficiency than HIV. This mechanism contributes to lower viremia, immune escape, and latency establishment [165,167,168].

Cell-to-cell virus transmission mainly involves T lymphocytes but has also been observed in dendritic cells and macrophages [169].

HTLV-1 is transmitted through body fluids, including blood (63% of transfusion-related cases), semen, and breast milk during breastfeeding.

According to the WHO, approximately 10 million people are infected with HTLV-1 globally, with endemic areas in Latin America, the Caribbean, Africa, and Japan (https://www.who.int/news-room/fact-sheets/detail/human-t-lymphotropic-virus-type-1, accessed on 19 October 2024; https://www.ecdc.europa.eu/sites/default/files/media/en/publications/Publications/geographical-distribution-areas-high-prevalence-HTLV1.pdf, accessed on 6 November 2024).

HTLV-1 was the first oncogenic human retrovirus discovered and is mainly associated with adult T-cell leukemia/lymphoma (ATL) and HTLV-1-associated myelopathy/tropical spastic paraparesis (HAM/TSP) [170]. Common clinical symptoms include lymphadenopathy, hepatosplenomegaly, and hypercalcemia. HAM/TSP, affecting 0.18–1.8% of individuals with infection, is a chronic inflammatory disease of the central nervous system that causes progressive spastic weakness in the lower limbs, sensory disturbances, and bowel/bladder dysfunction [171].

Although most individuals with HTLV-1 infection are asymptomatic, approximately 5% develop ATL over their lifetime. Among ATL clinical subtypes, the acute form (55–60%) is the most aggressive, followed by lymphomatous (20%), chronic (20%), and smoldering (5%) [172,173]. The median survival times are 8, 10, 35, and 55 months, respectively [174].

In patients with ATL, increased viremia and reduced levels of blood Tax protein are associated with a higher risk of developing severe ATL [175]. Two important players in viral replication and oncogenesis are Tax and HBZ (HTLV-1 basic leucine zipper factor) [176]. The U3 region of the LTR contains Tax responsive element 1 (TRE-1), which serves as a binding site for the viral oncoprotein Tax. Tax binds a sequence within TRE-1, the viral cAMP response element (vCRE), recognized by the cellular CRE binding protein (CREB) [177]. Tax binding stabilizes CREB at viral promoters, enhancing gene expression and shifting the virus from latency to active replication [178]. Tax can deregulate cell cycle regulation by inhibiting p53 and pRb and lead to chromosomal instability by impairing cellular DNA repair mechanisms, both hallmarks of cancer [177]. Moreover, Tax activates several key cellular signal transductions involved in chromatin remodeling, cell proliferation, and survival, regulating the immune response, such as the CREB, AP-1, and NF-κB pathways, and promoting tumorigenesis [179].

Unlike HIV, HTLV-1, in order to sustain the replication cycle, must activate T-cells during the early stages of infection [180]. Specifically, the Tax protein of HTLV-1 promotes the self-activation of infected naïve T lymphocytes to T-regulatory cells, which, being excessive, lead to clonal expansion underlying transformation into ATL cells [180].

However, Tax expression is often absent in later ATL stages due to epigenetic silencing or mutations in the promoter within the 5′LTR [181]. Primary ATL cells of leukemia patients express very low levels of transcript and Tax protein, resulting in reduced levels of NF-κB activation [181].

The remarkable reduction in or loss of Tax expression in ATL cells has been attributed to epigenetic silencing through methylation or accumulation of mutations, including deletions, in the promoter located within the 5′LTR [181]. According to the ATL pathogenesis model, the strong immunogenicity of Tax and its induction of apoptosis lead to the rapid elimination of Tax-expressing cells by the immune system, selecting for the persistence of Tax leukemic clones with a suboptimal expression of the viral protein, which are more resistant to immune surveillance and allow Tax-mediated immortalization [181,182].

Unlike Tax, which is not detected in most ATL cells, HTLV-1 B-zip factor (HBZ) is a transcriptional repressor expressed in all cells and acts opposite to Tax [183]. HBZ mRNA has been detected in all ATL patients, whereas Tax transcripts are present only in 30–40% of cases [184].

This phenomenon can be explained by the interplay between HBZ transcripts and proteins: HBZ is transcribed in the opposite direction to Tax, allowing HBZ mRNA to act as an antisense molecule that silences Tax expression. In addition, HBZ acts as a negative transcriptional regulator by interacting with CREB and preventing the formation of the Tax/CBP/CREB complex on the 5′LTR, thus reducing the expression of viral transcripts [183].

In summary, the interaction between Tax and HBZ is crucial in balancing viral replication, latency, and oncogenesis in HTLV-1 infection. HBZ supports the oncogenic potential of the virus by promoting the survival of infected cells during latency, inhibiting apoptosis and senescence. Even when Tax expression is low or absent, HBZ can still sustain the transformed phenotype of infected T-cells and prevent their elimination by the immune system.

Another important role in this activation is played by the viral accessory protein p12, which modulates the signaling transduction pathways required for T lymphocyte activation, such as NF-κB, AP-1, and Nuclear Factor of Activated T-cells (NFAT) [185].

The cellular proteins CD25, FOXP3, IL-2 receptor, and HLA class II represent hallmarks of activation that are progressively induced and enhanced in HTLV-1 infection and transformation [186].

In addition, HTLV can remain latent after integration into T-cells, establishing a persistent infection that allows its long-term expression even in the absence of intense immune activation [187].

### 3.2. Human Immunodeficiency Virus

The Human Immunodeficiency Virus (HIV), belonging to the *Lentivirus* genus, has a spheroidal morphology and is an envelope virus of about 100–110 nm in diameter. The capsid has a truncated cone shape and encloses two copies of a genome of 9.75 kb. When retrotranscribed to DNA, the proviral genome contains two long terminal repeat (LTR) sequences; three genes, gag, pol, and env; and six genes coding for two regulatory proteins, Trans-activating (Tat) and Regulator of virion (Rev), and four accessory proteins, Negative factor (Nef), Virion infectivity factor (Vif), Viral protein R (Vpr), and Viral protein unique (Vpu). The Gag, Pol, and Env genes each code for a polypeptide precursor proteolytically processed. Gag will give rise to the capsid protein (CA, p24), outer core membrane (MA, p17), nucleocapsid (NC, p7), and a smaller protein (p6) [188].

Reverse transcriptase (RT, p51) plus RNase H (p15), integrase (IN, p32), and protease (PR, p12) were obtained by cleavage of the polypeptide Pol [189]. Two envelope glycoproteins were derived from the Env polypeptide: surface protein (gp120) and transmembrane protein (gp41) were used as VAP and fusion protein, respectively [190].

LTR sequences are very important for the insertion of the viral genome into the genome of the host cell. Unlike HTLV-1, the virally encoded oncoprotein is not present in HIV-1.

HIV transmission occurs via parenteral (blood transfusion, injection with infected fomites, surgery, and dental procedures), sexual (seminal fluids), and perinatal [191].

HIV infects cluster of differentiation 4 (CD4+) cells of the immune system, such as T lymphocytes, monocyte–macrophages, and dendritic cells. For infection, HIV requires, in addition to CD4 as a receptor, a co-receptor represented by CCR5 (C-C chemokine receptor type 5) and CXCR4 (C-X-C chemokine receptor type 4), which defines a monocyte–macrophage (R5) or lymphocyte (X4) tropism of the virus, respectively [162].

In the early stages, the infection is mainly carried by R5-tropic viruses, in which no significant CD4+ T lymphocyte depletion is observed, which is typical of acquired immunodeficiency syndrome (AIDS) in the late stages of the disease. As a result of immune selective pressure, R5X4 dual-tropic viruses are selected, in which the co-receptor at CXCR4 is also utilized. The complete switch from R5 to X4 tropic strains is rather rare [192].

HIV-1, despite lacking specific proteins and a dedicated program to regulate the establishment and maintenance of latency, is able to determine latent infections in memory CD4+ T-cells and other reservoir cells. The molecular trigger for the switch between viral production and latency is the viral accessory protein Tat; notably, the absence of Tat is associated with the viral latency phase [193].

To escape from the immune system, viral particles do not release outside the cells, but the virus adopts a strategy of cell-to-cell transmission known as virological synapses. Moreover, this mechanism facilitates both CD4+ T-cell latency establishment and virus trafficking cell-to-cell, contributing to the persistence of infection and disease progression [194].

The virological synapse is a dynamic structure formed when, during viral particle assembly, gp120 in the plasma membrane of the infected (donor) cell binds to the CD4 receptor of un-infected (recipient) T-cells. This interaction also recruits gp41 and cellular proteins, such as CCR5 or CXCR4 co-receptors, and adhesion molecules (LFA-1 and ICAM-1) to the surface of the infected cell, stabilizing the synapse and facilitating efficient viral transfer across cells. The binding of gp120 to CD4 induces conformational changes in gp41, which mediates the fusion of the membranes of infected and non-infected cells, called “fusion from within”, and the viral core, including RNA and other components, is directly transferred into the target cell, allowing efficient viral transmission without the virus being released into the extracellular space [169].

Rarely, virological synapsis involves a process of endocytosis in which the HIV-1 immature particles present in the extracellular space can be internalized into the target T-cells via dynamin- and clathrin-dependent vesicles followed by the maturation of the viral particles in endosomal compartments. There will be partial intracellular morphogenesis, unlike what normally occurs in the virus replication cycle, where the viral particles complete maturation in the extracellular environment. In fact, the viral particle fuses the envelope with the endosome membrane, releasing the nucleocapsid and associated viral protein into the cytoplasm [169].

Since the beginning of the epidemic, the WHO estimates that approximately 88.4 million (range 71.3–112.8 million) people worldwide are living with HIV and that approximately 42.3 million (range 35.7–51.1 million) have died from HIV-related AIDS complications (https://www.who.int/data/gho/data/themes/hiv-aids, accessed on 26 October 2024). Based on these epidemiological statistics, we can consider HIV as a silent pandemic.

It has been demonstrated that mutations specifically interfering with the integration process block viral replication not only in HIV but also in other retroviruses, such as the spleen necrosis virus (SNV) and murine leukemia virus (MLV) [195,196,197,198].

In HIV, as in other retroviruses, the viral integration process is crucial for two main reasons:

(1) **Episomal retroviral DNA is incapable of autonomous replication**. Proviral DNA can only be duplicated and transmitted as part of the host genome. Unlike other retroviruses that replicate only in dividing cells, HIV replicates in resting lymphocytes due to its ability to pass the nuclear membrane via the pre-integration complex (PIC). The PIC is composed of proteins from the infecting virion (e.g., Vpr, RT, and integrase) and cellular proteins. As a result, HIV replication does not depend on proliferating B lymphocytes and rarely associates with oncogenic processes, except in rare cases discussed later. Conversely, retroviruses like HTLV-1 lack the pre-integration complex and require actively dividing lymphocytes to allow proviral DNA to access the host genome by bypassing the nuclear membrane. Consequently, such retroviruses are often associated with the onset of lymphoma.
(2) **Integration stabilizes viral DNA.** During acute infection, non-integrated viral DNA in infected cells degrades shortly after formation, leading to the resolution of infection in the absence of integration into the host genome [199,200,201].

**Figure 3:**
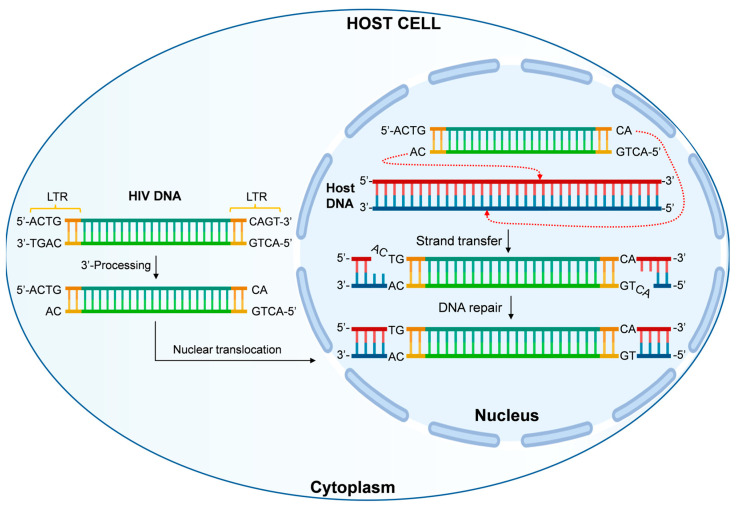
HIV integration process. After the reverse transcription of HIV RNA, the integrase enzyme performs 3′-processing, leaving the CA dinucleotide extending at both 5′ ends of the HIV genome. Following nuclear translocation, HIV strand transfer occurs, where integrase mediates the cleavage and insertion of viral DNA into the host genome. The CA dinucleotide at the 5′ ends is excised, and the resulting gaps are repaired by cellular genome repair mechanisms, completing the HIV integration process.

Furthermore, integration is critical for efficient transcription of the viral genome, synthesis of genomic RNA for encapsidation into new viral particles, and production of viral proteins. Like other retroviruses, the integrated HIV proviral DNA is identical to the linear DNA synthesized from the viral RNA genome, except for the loss of two bases at the 5′ and 3′ ends (CA dinucleotide). Viral DNA integration into the host genome always occurs via a consistent mechanism, accompanied by a short duplication (4–6 bp) of the target sequence flanking the integrated provirus (Figure 3) [202,203].

Once integration is completed, the process is irreversible. However, unlike retroviruses such as MLV and ALV, HIV integration appears to be neither mutagenic nor oncogenic. As with transposons and retrotransposons, the retroviruses and HIV linear DNA ends must interact with integrase, the enzyme catalyzing the integration. The integrase–viral DNA complex, known as the intasome, is associated with the pre-integration complex [204,205,206]. At the integration site, only the 3′ ends of the viral DNA attach to the target DNA, while the 5′ ends extend two nucleotides beyond the CA dinucleotide on the joined strands [205,207]. Linear DNA is the predominant form in infected cells and accumulates in the cytoplasm. Other circular DNA forms accumulate in the nucleus, representing non-integrated viral DNA [208]. These three forms are (1) circular DNA with a single LTR; (2) circular DNA with two LTRs (2-LTR circles), likely formed by ligation of linear DNA ends, sometimes with nucleotides deletions [209]; and (3) auto-integrated DNA, resulting from intramolecular integration events leading to rearranged circular DNA [210].

Several factors can impair integration, such as incomplete reverse transcription or defective pre-integration complex assembly due to restriction factors like APOBEC3G (apolipoprotein mRNA-editing enzyme catalytic polypeptide-like 3G) or a cytidine deaminase that induces hypermutation in viral DNA, countered by the viral protein Vif (Viral infectivity factor) [211].

In macrophages, circular forms of HIV DNA can persist for up to two months but appear to contribute minimally to viral persistence [212].

Non-integrated DNA forms do not participate in the viral integration process or replication but instead appear to represent dead-end byproducts resulting from defective mutants [213]. These molecules are often transcriptionally silenced within the nucleus, a phenomenon referred to as viral pre-integration latency [198].

Furthermore, HIV integration occurs efficiently in both dividing and non-dividing cells [214,215,216]. Although integration may cause host genome damage, it is rapidly repaired [217].

Integrated proviral DNA can remain latent through interactions with histones and other factors, such as histone chaperones (Chaf1a/1b), SUMOylation factors, and DNA methylation [218]. Latent silencing provides advantages: (1) the lack of viral protein synthesis prevents the activation of the immune system, avoiding the elimination of the infected cells and establishing a state of chronic latent infection, and (2) it complicates the development of antiviral drugs aimed at eradicating the infection. Latent HIV reservoirs, where replicative competence persists despite the provirus being in a stable quiescent state, primarily reside in CD4+ memory T-cells, although macrophages and monocytes can also harbor latent infections [219]. As demonstrated by Swiggard, CD4+ memory T-cells, not in an active replication phase, can be infected by HIV [220].

Following primary infection, the number of latently infected cells is estimated to be around 10⁸, with approximately 10% capable of replication [221,222].

Transcriptional activation depends on the integration site, with euchromatic regions favoring transcription. Integration in these regions induces the rapid activation of the transcriptional process and a decrease in repressive factors, like histones. Viral proteins, such as Tat, and the interaction between cellular factors and 5′ LTR regions, which contain multiple binding sites for transcriptional regulators, are involved in the activation process. Transcription can also be stimulated by external signals, including cytokines and antigen exposure.

Although HIV has so far not been considered a virus capable of triggering tumors, it has been observed that some accessory proteins could promote carcinogenesis. Although they can also be detected in the plasma of HIV low-viremia patients, much remains to be investigated [223]. For example, the Tat protein plays a key role in oncogenesis, for example, by promoting MYC-IGH (MYC–Immunoglobulin Heavy Chain) colocalization, which could explain the high risk of chromosome translocations observed in aggressive lymphomas. Tat was detected in BL at a significantly higher rate in positive than HIV-negative patients [223,224].

## 4. Discussion and Conclusions

Persistence is established after primary infection following a fine balance between virus and host that ensures survival. It is a strategy used by many viruses to evade eradication by deregulating the immune system, ensuring permanence and transmission within the host to optimize viral fitness.

Long-lasting persistence includes low-level viral replication, genetic and epigenetic modifications, environmental factors (e.g., smoke, alcohol), and, especially, viral genome maintenance and reliance on viral oncoprotein production [225,226].

Oncogenesis usually occurs when a persistent viral infection is associated with the impairment of mechanisms of cell growth, apoptosis, and DNA repair [227,228,229]. In human oncogenic viruses, the genome is found in an episomal form or integrated into the host’s DNA [230]. The process of genome integration occasionally occurs at different stages of infection (e.g., HBV), during the chronic phase of infection (e.g., HPV, EBV), or otherwise may be an essential part of the viral life cycle, as seen in retroviruses (HIV, HTLV-1) [230,231]. Notably, integration is an irreversible process. Several models have been proposed to describe the genome integration process, including non-homologous recombination, looping, and microhomology models. Integration can occur randomly or at specific genomic sites, often leading to the destabilization of the genome. In some cases, integration results in the loss of genomic regions or impairs the regulation of oncogene and/or oncosuppressor expression, contributing to tumor development and often the exacerbation of inflammation. Otherwise, the persistence of extrachromosomal viral genomes usually promotes oncogenesis via the constitutive expression of viral proteins that interfere with cell cycle regulation [232].

For viruses such as HPV, HBV, and EBV, viral genome integration into the host enhances the ability to establish persistence and progression towards the neoplastic transformation of the infected cell with a marked increase in the risk of developing tumors and/or greater aggressiveness [156,233,234,235]. In HPV infections, genome integration is common in high-risk (HR) strains and results in the disruption of the E2 gene, and this loss impairs the regulation of oncogene expression, mainly E6 and E7 oncoproteins, contributing to carcinogenesis [58].

The episomal form can induce both persistence and cellular transformation so that integration does not occur in all HPV infections, and these may resolve through the host cell’s immune response [236].

In the case of HBV, genome integration is a sporadic event (<1% of infected hepatocytes) occurring at any stage of the virus life cycle, in both the acute and chronic phases, and transformation into hepatocarcinoma is not always accompanied by the integration of the host genome. However, other factors such as alcohol, smoking, and steroids may promote the carcinogenic process [155]. In particular, the integration site is essential, with a greater impact on oncogenic transformation when integration occurs in regions flanking genes encoding cell cycle regulatory proteins [15,89].

Like HPV, EBV has a DNA genome that resides in the host cell’s nucleus, primarily as extrachromosomal episomes, with a smaller percentage integrated into the human genome. Unlike other oncogenic viruses such as HPV and HBV, EBV does not have specific integration hotspots within the DNA genome. However, in Burkitt’s lymphoma (BL) cells, integrations occur at multiple sites in a non-random pattern. These integrations often occur in fragile regions during DNA repair and, when they occur in the proximity of tumor suppressor genes, may increase the risk of tumor development. Moreover, unlike HPV and HBV, it presents specific latency programs that are associated with different tumor forms [129].

Retroviruses, to which HIV and HTLV-1 belong, are the only viruses that require an obligatory integration step for their replication cycle and permanence in the host cell. They encode for their integrase, indispensable for the integration process, and only the integrated forms are productive. In contrast, the other forms cannot give rise to viral progeny.

The proviral DNA of retroviruses produces all viral proteins, whereas in the case of HBV and HPV, viruses in the process of integration can lose the functionality of proteins such as E2 and E5 (common), E6 and E7 (rare) (HPV), or pre-S1 (HBV) [58,237].

The significant difference between HTLV-1 and HIV-1 that underlies the opposed pathology that they cause concerns the outcome of integration into a host cell’s genome. Specifically, in HTLV-1, proviral integration results in the expression of the viral oncoprotein Tax, responsible for the proliferation of T lymphocytes, potentially causing leukemia and lymphoma. Instead, in HIV, genome integration causes an immune deficit of T lymphocytes, which leads to AIDS.

In conclusion, the persistence of viral infection, both in extrachromosomal (episome) and integrated forms, plays a crucial role in viral oncogenesis. Viruses that integrate their genomes, such as HIV, HTLV-1, HPV, HBV, and EBV, use different mechanisms to elude the immune system, promote chronic inflammation, and deregulate cellular processes and pathways. Genome integration into host DNA commonly results in a higher risk of cancer progression, as it leads to long-lasting viral persistence, oncogene activation, and genomic instability. On the other hand, viruses such as HPV and EBV can also induce cancer when they exist in an episomal form, although the risk is generally lower than integration.

On the other hand, in the context of viral carcinogenesis, the “hit-and-run” theory offers an explanation for how certain viruses (hits) can contribute to cancer development by integrating into the host genome and/or not persisting (runs) for a long time in the host. According to this theory, the virus causes genetic changes, epigenetics modifications, or deregulation of the cell cycle or signal transduction pathways in the host cell. After this initial “effect”, the virus no longer needs to remain in the host cell or persist for an extended period because the cell that has been “modified” can initiate the program towards malignant transformation on its own [238].

This theory is particularly relevant for understanding how viruses such as HPV, HBV, and EBV, which do not necessarily require permanent integration into the host genome, contribute to cancer development, even when the virus is not detected in tumor cells.

In the early stages, viral genome integration may occur. However, depending on the type of virus and tumor, this integration can be lost during clonal expansion or through the eradication of the infected cell by the host immune system. Emerging platforms now enable the analysis of clonal integration at the virometric level, helping uncover the viral origins of cancer and detect integrations even at early stages [239].

Understanding the integration mechanisms and their strict synergy relationship with viral persistence and oncogenesis lays the groundwork for developing new antiviral therapeutic strategies to reduce viral load and virus permanence in the host, lowering the risk of developing tumors.

## Data Availability

Not applicable.
